# Neuronal Ceroid Lipofuscinosis in a Mixed-Breed Dog with a Splice Site Variant in *CLN6*

**DOI:** 10.3390/genes15060661

**Published:** 2024-05-23

**Authors:** Tendai Mhlanga-Mutangadura, Garrett Bullock, Sofia Cerda-Gonzalez, Martin L. Katz

**Affiliations:** 1 Canine Genetics Laboratory, Department of Veterinary Pathobiology, College of Veterinary Medicine, University of Missouri, Columbia, MO 65211, USA; tendai@missouri.edu (T.M.-M.); gebkd2@missouri.edu (G.B.); 2 MedVet Chicago, Chicago, IL 60618, USA; sofia.cerda-gonzalez@medvet.com; 3 Neurodegenerative Diseases Research Laboratory, Department of Ophthalmology, School of Medicine, University of Missouri, Columbia, MO 65212, USA

**Keywords:** neurodegeneration, lysosomal storage, mitochondrial subunit c protein, lipofuscin, fluorescence, whole genome sequencing, electron microscopy

## Abstract

A 23-month-old neutered male dog of unknown ancestry presented with a history of progressive neurological signs that included anxiety, cognitive impairment, tremors, seizure activity, ataxia, and pronounced visual impairment. The clinical signs were accompanied by global brain atrophy. Due to progression in the severity of disease signs, the dog was euthanized at 26 months of age. An examination of the tissues collected at necropsy revealed dramatic intracellular accumulations of autofluorescent inclusions in the brain, retina, and cardiac muscle. The inclusions were immunopositive for subunit c of mitochondrial ATP synthase, and their ultrastructural appearances were similar to those of lysosomal storage bodies that accumulate in some neuronal ceroid lipofuscinosis (NCL) diseases. The dog also exhibited widespread neuroinflammation. Based on these findings, the dog was deemed likely to have suffered from a form of NCL. A whole genome sequence analysis of the proband’s DNA revealed a homozygous C to T substitution that altered the intron 3–exon 4 splice site of *CLN6*. Other mutations in *CLN6* cause NCL diseases in humans and animals, including dogs. The CLN6 protein was undetectable with immunolabeling in the tissues of the proband. Based on the clinical history, fluorescence and electron-microscopy, immunohistochemistry, and molecular genetic findings, the disorder in this dog was classified as an NCL resulting from the absence of the CLN6 protein. Screening the dog’s genome for a panel of breed-specific polymorphisms indicated that its ancestry included numerous breeds, with no single breed predominating. This suggests that the *CLN6* disease variant is likely to be present in other mixed-breed dogs and at least some ancestral breeds, although it is likely to be rare since other cases have not been reported to date.

## 1. Introduction

Among the most common hereditary neurodegenerative diseases in dogs are neuronal ceroid lipofuscinoses (NCLs) [1]. Mutations in at least 13 genes have been associated with different forms of NCL in human subjects [2,3]. Progressive neurodegenerative disorders in dogs have been associated with mutations in canine orthologs of the majority of these genes [1]. NCLs are lysosomal storage disorders that have been distinguished from other lysosomal storage diseases through the combination of phenotypic features consisting of predominantly neurological signs that progress over time, atrophy of the central nervous system, and a characteristic autofluorescence of the lysosomal storage bodies [4]. In addition to the 13 genes that have been associated with NCLs by consensus among many researchers, other hereditary disorders in dogs with NCL-like phenotypes have been described that result from mutations in genes that have not been associated with NCL in human subjects. Among these genes are *ARSG* and *CNP* [5,6,7]. For dogs that exhibit the characteristic signs of NCL, a whole genome sequence (WGS) analysis can be used to determine whether the disorder is associated with a mutation in any of these genes. In this study, a dog that exhibited progressive neurological signs suggestive of NCL was euthanized due to disease progression, the tissues were examined for NCL-like pathology, and a WGS analysis was performed to determine whether the disorder was associated with a mutation in any of the genes previously associated with NCL.

## 2. Materials and Methods

**Figure 1:**
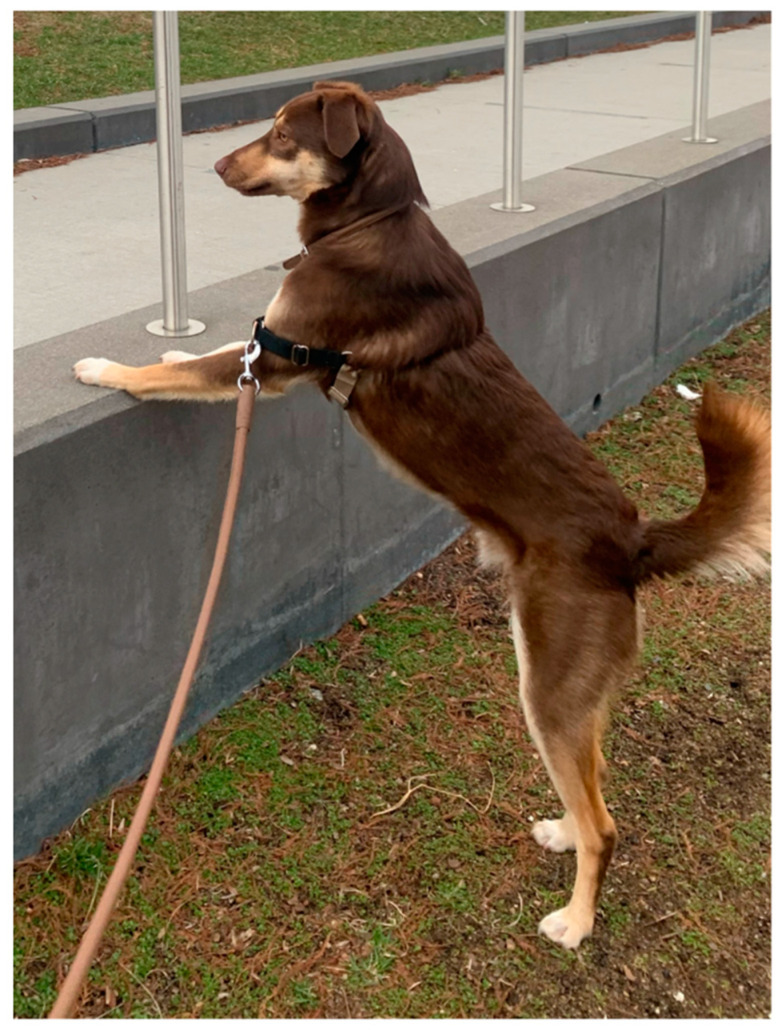
A photograph of the proband about the time of onset of neurological disease signs.

**Figure 2:**
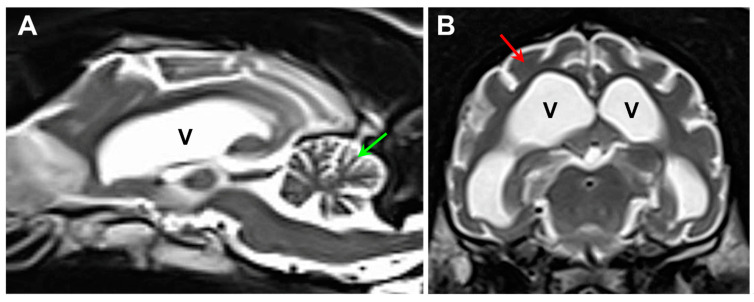
Sagittal (**A**) and axial (**B**) T2-weighted MR images of the proband’s brain. The ventricular system (V) was dramatically enlarged, and there was a pronounced shrinkage of the parenchyma of both the cerebellum (green arrow in (**A**)) and the cerebral cortex (red arrow in (**B**)).

A 23-month-old neutered male dog of unknown ancestry (Figure 1) presented after a 4-month history of progressive neurological signs that included anxiety, cognitive impairment, tremors, seizure activity, ataxia, incoordination, and pronounced visual impairment. Marked diffuse brain atrophy was documented with magnetic resonance imaging (Figure 2). The dog was euthanized at approximately 26 months of age due to the progression of the neurological signs. Following euthanasia, the eyes, brain, and heart ventricular wall were collected and preserved with aldehyde fixatives for light and electron microscopy, as described previously [8]. “Immuno Fix” consisting of 3.5% paraformaldehyde, 0.05% glutaraldehyde, 120 mM sodium cacodylate, and 1 mM calcium chloride with pH 7.4 was used for the light microscopy and immunohistochemistry samples. “EM Fix” consisting of 2% glutaraldehyde, 1.12% paraformaldehyde, and 120 mM sodium cacodylate with pH 7.4 was used for electron microscopy samples. Unstained cryostat sections of the retina, cerebral cortex, cerebellar cortex, and cardiac muscle fixed for light microscopy were examined for lipofuscin-like autofluorescence [7]. Slices of the same tissues fixed in “Immuno Fix” were embedded in paraffin. Sections of the paraffin-embedded samples were immunostained using Abcam (Cambridge, UK) anti-mitochondrial ATP synthase subunit c primary antibody (cat. no. ab180149, dilution 1:100), Agilent Dako (Agilent Technologies, Santa Clara, CA, USA), anti-GFAP primary antibody (cat. no. Z0334, dilution 1:200), Fujifilm Wako (Fujifilm North America, Lousiville, KY, USA), anti-Iba1 primary antibody (cat. no. 019-19741, dilution 1:100), and Abcam anti-CLN6 antibody (cat. no. ab272678, dilution 1:50). Antigen retrieval and immunostaining was performed as described previously [7,9]. Paraffin sections of immuno-fixed brain samples from a 12-month-old Shiba Inu that was euthanized due to seizures were also immunolabeled with the anti-CLN6 antibody. Pieces of the same tissues fixed in “EM Fix” were post-fixed with osmium tetroxide and embedded in epoxy resin. Thin sections of the latter samples were examined with transmission electron microscopy using a JEOL JEM-1400 microscope equipped with a Gatan digital camera.

Genomic DNA was prepared from EDTA-anticoagulated blood of the proband [10] and was submitted to the University of Missouri Genomics Technology Core Facility for library preparation and 2 × 150 bp paired-end sequencing on their Illumina NovaSeq 6000 sequencer. An alignment of the sequence reads to the current canine reference genome assembly (Dog10K_Boxer_Tasha) and variant calling was initially performed using the OVarFlow 2 workflow software [11]. In addition, a previously described data-processing pipeline was used to align the sequence reads to the same genome assembly and to analyze them with Ensembl annotation in conjunction with reads from 334 other whole genome sequences generated at the University of Missouri Canine Genetics Laboratory and deposited in the NCBI Sequence Read Archive (SRA) [7]. The SRA BioSample identifier for the proband is SAMN39309507. The SRA BioSample identifiers of the remaining whole genome sequences used in this analysis were reported previously [6]. The amino acid positions for canine *CLN6* were numbered according to XP_038298694.1 (NCBI).

An allelic discrimination assay was used to genotype individual dogs for a candidate variant at position 32,185,406 on chromosome 30. For this assay, the sequences of the PCR primers were 5′-AAGGTGATGATGCTGACGTAGATC-3′ and 5′-GCCACGGCCTCCCT-3′. The competing probes’ sequences were 5′-VIC-CTGACCTCAGCTCATC-NFQ-3′ (reference allele) and 5′-FAM-CTGACCTCAACTCATC-NFQ-3′ (variant allele). This assay was used to genotype 13 mixed-breed dogs with neurological disorders of unknown etiology represented in our DNA archive.

A DNA sample from the proband was submitted to Wisdom Health (Portland, OR, USA) for genotyping with the Wisdom Panel Premium. This panel includes genotyping for variants that indicate a dog’s breed composition. It also includes genotyping for over 235 mutations that have been shown to underlie hereditary disorders in dogs. Among the disease variants in the test panel are NCL-causing mutations in *PPT1*, *ATP13A2*, *MFSD8*, and *CLN8*.

## 3. Results

### 3.1. Microscopic Findings

**Figure 3:**
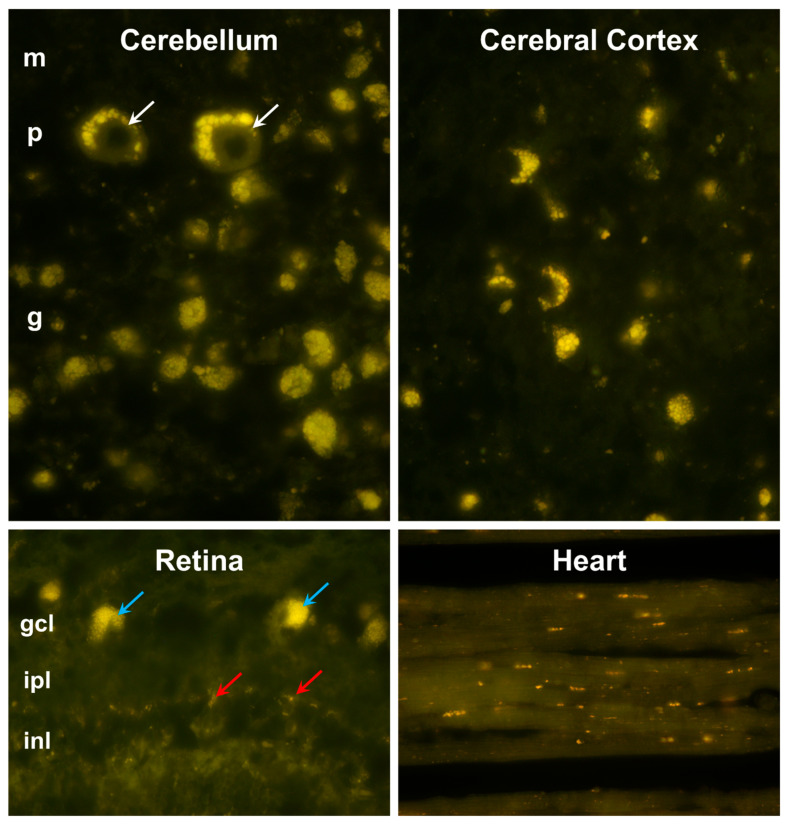
Fluorescence micrographs of unstained cryostat sections of the cerebellar cortex, cerebral cortex occipital lobe, retina, and cardiac muscle. In the cerebellum, aggregates of autofluorscent inclusions were present in cells of the molecular (m), Purkinje (p), and granule cell (g) layers, with particularly large granules in the Purkinje cells (white arrows). In the retina, accumulations of these inclusions were most prominent in the ganglion cells (blue arrows), with small individual autofluorescent granules in other layers of the retina (red arrows). The retinal layers shown include the ganglion cell layer (gcl), inner plexiform layer (ipl), and inner nuclear layer (inl). In the cerebral cortex, autofluorescent storage material was present in cells throughout the gray matter. In cardiac muscle, the autofluorescent granules occurred in linear arrays within the muscle fibers parallel to the long axes of these fibers.

The proband exhibited pronounced accumulations of autofluorescent inclusions in the cerebellum, cerebral cortex, retina, and cardiac muscle (Figure 3). These inclusions displayed yellow to orange emissions when illuminated with blue light. Cells containing large aggregates of these inclusions were present throughout the cerebellar and cerebral cortexes. In the retina, large aggregates of the autofluorescent inclusions were present only in the ganglion cells, while individual autofluorescent granules were scattered among cells in the other layers of the retina. In the cardiac muscle, autofluorescent inclusions were clustered in linear arrays within the muscle fibers.

**Figure 4:**
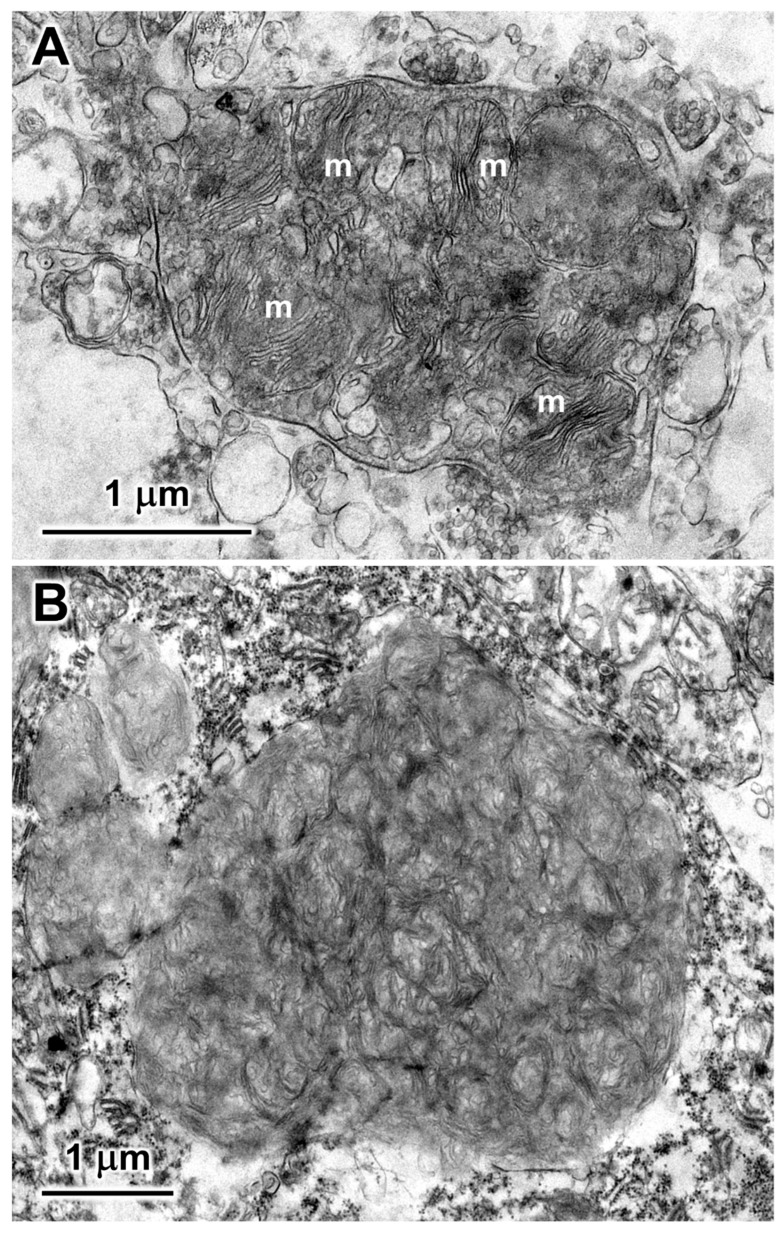
Electron micrographs of representative storage bodies in the cells of the cerebellar cortex. The storage body in (**A**) contains a number of inclusions that appear to be derived from mitochondria (m). In (**B**), the organization of the membrane-like contents of the storage body is more heterogeneous.

**Figure 5:**
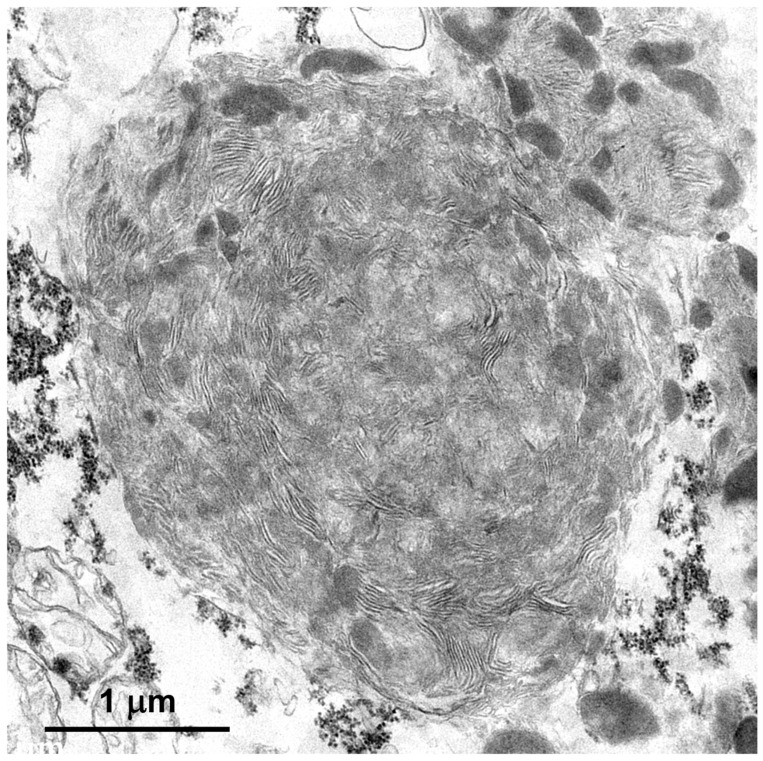
An electron micrograph of a disease-related storage body in a cerebral cortical neuron of the proband.

**Figure 6:**
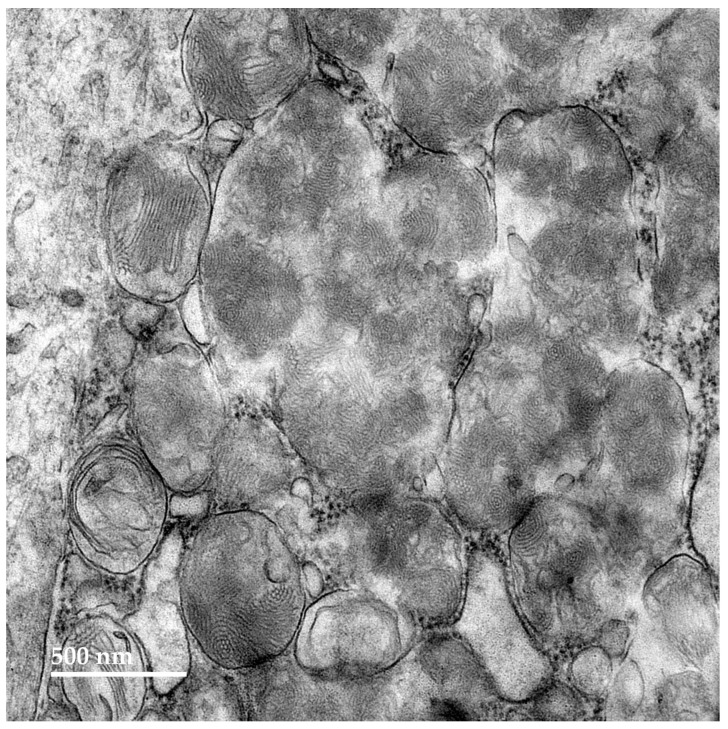
An electron micrograph of a cluster of disease-related storage bodies in a retinal ganglion cell of the proband.

**Figure 7:**
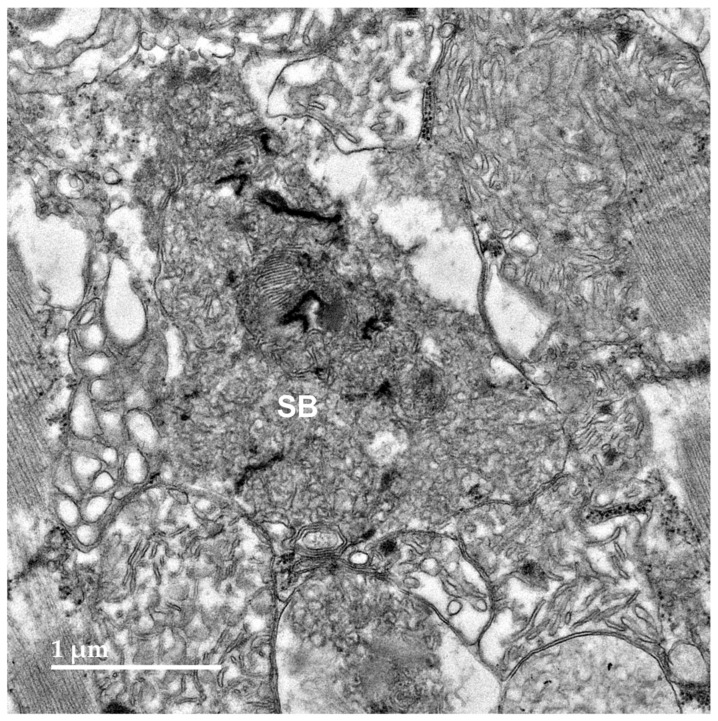
An electron micrograph of the disease-related storage body (SB) in the cardiac muscle of the proband.

**Figure 8:**
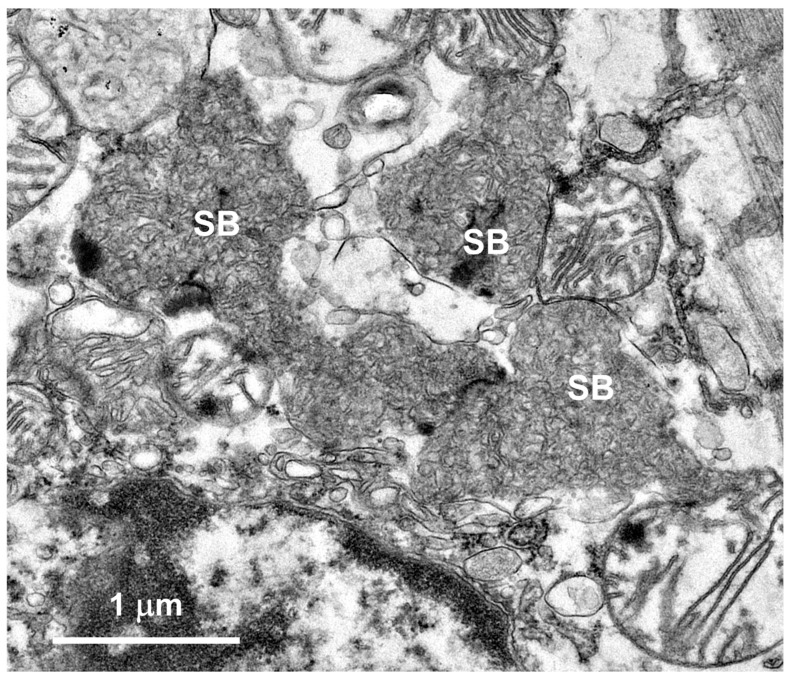
An electron micrograph of a cluster of disease-related storage bodies (SBs) in the cardiac muscle of the proband containing primarily vesicular structures.

**Figure 9:**
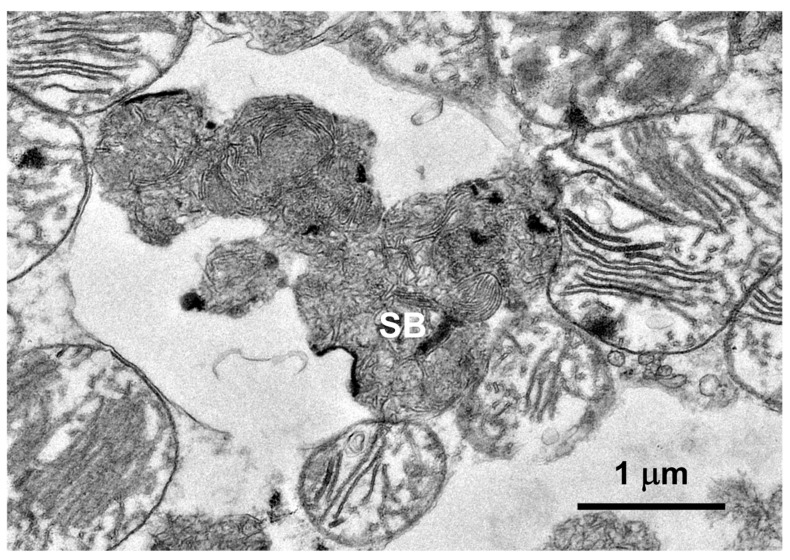
An electron micrograph of the disease-related storage body (SB) in the cardiac muscle of the proband containing stacks of membrane-like components.

Within cells of the cerebellar cortex, some of the disease-related intracellular inclusion bodies appeared to be autophagolysosomes that contained structures with morphological appearances suggesting that they were derived from mitochondria, as well as a heterogenous mixture of membrane-like components (Figure 4A). The majority of the inclusion bodies in the cerebellar cortex consisted of tightly packed membranous components (Figure 4B). In the cerebral cortex, the contents of the storage bodies consisted primarily of stacks of membrane-like components in random orientations (Figure 5). In the retinal ganglion cells, tightly packed clusters of storage bodies contained membrane-like components that were mostly arranged in fingerprint-like patterns (Figure 6). In the cardiac muscle¸ the storage bodies were clustered among groups of mitochondria that flanked many of the muscle fiber cell nuclei. The contents of the storage bodies consisted of tightly packed vesicular structures of similar size and stacks of membrane-like structures (Figure 7, Figure 8 and Figure 9).

**Figure 10:**
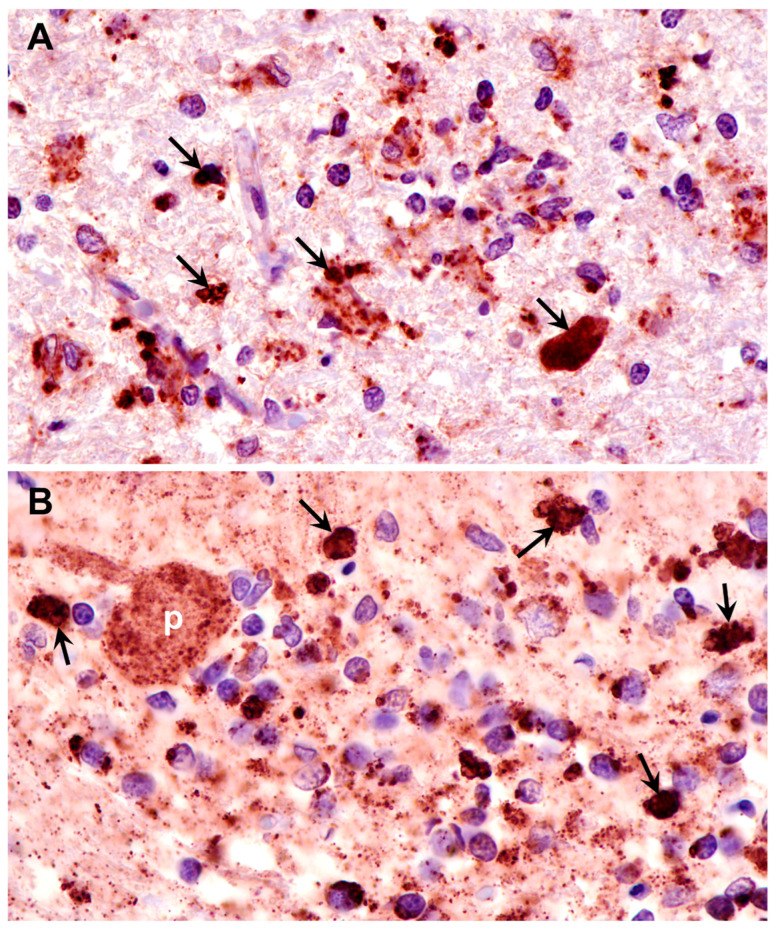
Paraffin sections of cerebral cortex gray matter (**A**) and cerebellar cortex (**B**) of the proband immunostained with an anti-subunit c antibody. Dark brown immunostain (arrows) was present in the numerous cells of both brain regions, including Purkinje cells (p) of the cerebellum.

**Figure 11:**
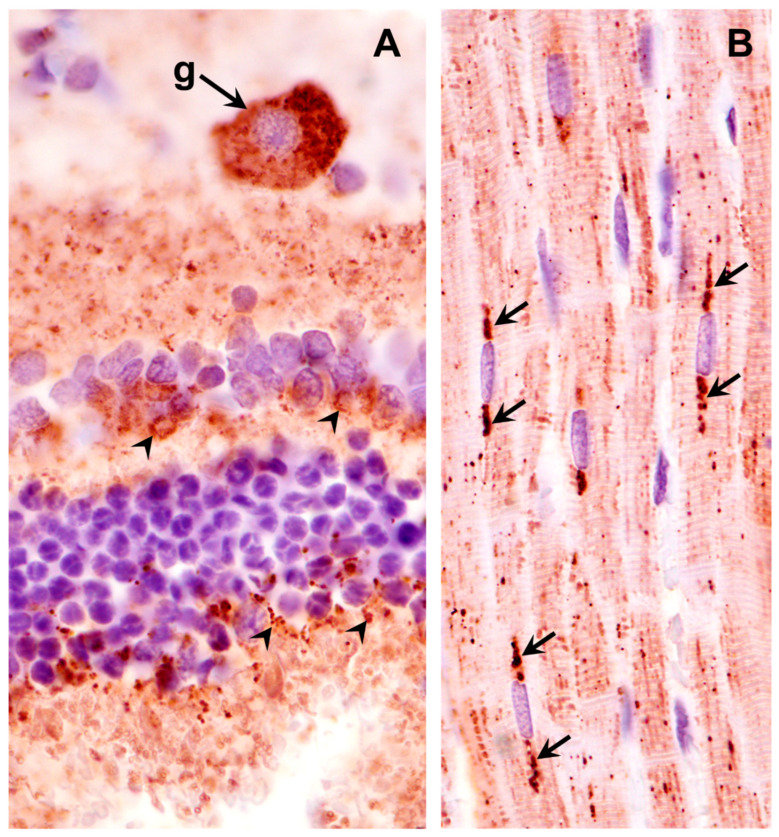
Paraffin sections of the retina (**A**) and cardiac muscle (**B**) of the proband immunostained with an anti-subunit c antibody. Dark brown subunit c immunostain (arrows and arrowheads) was present in retinal ganglion cells (g), scattered throughout the rest of the retina (arrowheads in (**A**)), and in rows of punctate inclusions flanking the nuclei of cardiac muscle fibers (arrows in (**B**)).

The subunit c protein of mitochondrial ATP-synthase is a major component of the lysosomal storage bodies that accumulate in many forms of NCL, including the form that results from *CLN6* mutations [12]. Sections of the brain, retina, and cardiac muscle of the proband showed substantial immunostaining of intracellular inclusions with an anti-subunit c antibody (Figure 10 and Figure 11). The localization of the immunostaining was the same as the localization of the autofluorescent inclusions in these tissues.

**Figure 12:**
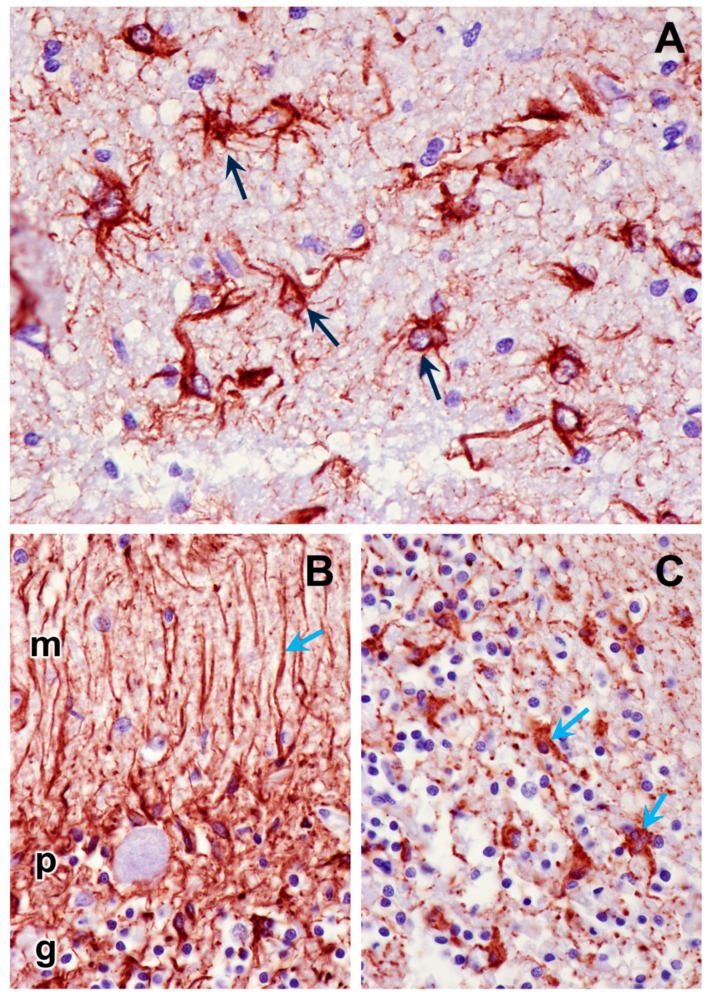
Paraffin sections of cerebral cortex gray matter (**A**) and cerebellar cortex (**B**,**C**) immunostained for GFAP localization (dark brown stain). The intensely stained cells (black arrows in cerebral cortex, blue arrows in cerebellar cortex) are activated astrocytes. The processes of the activated astrocytes extended throughout the molecular (m), Purkinje cell (p), and granular (g) layers of the cerebellar cortex (**B**). The granular layer of the cerebellar cortex is shown in (**C**).

**Figure 13:**
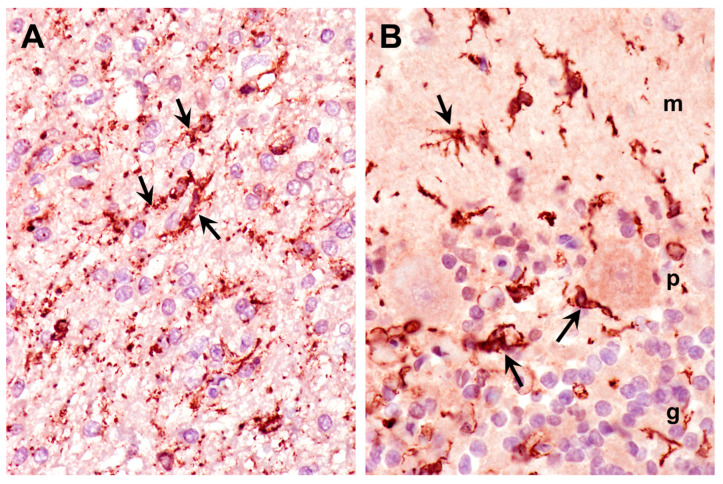
Paraffin sections of cerebral cortex gray matter (**A**) and cerebellar cortex (**B**) immunostained for Iba1 localization (dark brown stain). The intensely stained cells (arrows) are activated microglia. Activated microglia were present within the molecular (m), Purkinje cell (p), and granular (g) layers of the cerebellar cortex (**B**).

Neuroinflammation is often characterized by glial activation. Activated astrocytes, detected with GFAP immunolabeling, were abundant throughout the cerebral cortex and cerebellum of the proband (Figure 12). The proband also exhibited abundant activated microglia, detected with Iba1 immunolabeling, in the cerebral cortex and cerebellum (Figure 13).

### 3.2. Molecular Genetic Findings

The proband was adopted from a rescue service, and no pedigree information was available. Wisdom Panel genotyping was performed to characterize the breed background of the proband. Based on the dog’s pattern of DNA sequence variants, its ancestry was found to include over 25 breeds, with no one breed accounting for more than 10% of the genome. Thus, it was unlikely that the proband’s disorder resulted from known canine NCL mutations that are almost all either breed-specific or shared by a few similar breeds. The genotyping analysis indicated that the degree of heterozygosity in the proband was 40%, which is typical for mixed-breed dogs and higher than most purebred dogs.

**Figure 14:**
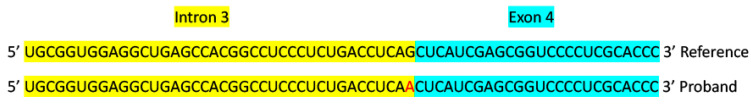
Predicted partial *CLN6* pre-mRNA sequences from the reference canine genome and the genome sequence of the proband showing the location of the G>A substitution at the 3′ end of intron 3.

**Figure 15:**
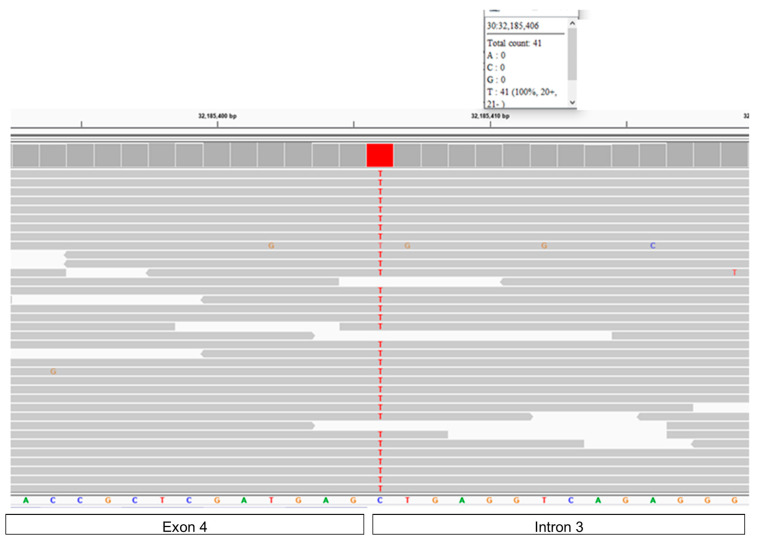
Reads of the proband’s whole genome sequence aligned to the reference sequence in the vicinity of position 32,185,406 on chromosome 30 as viewed with the Integrative Genomics Viewer. The T variant is highlighted in red. The reference sequence is below the series of gray bars, each of which represents a read from the proband’s whole genome sequence. Except where indicated with a nucleotide letter designation within a gray bar, the sequence of each read matches that of the reference sequence. The bars at the bottom of the figure indicate the regions of the reference sequence corresponding to the region of *CLN6* surrounding the intron 3–exon 4 boundary.

To elucidate the potential molecular genetic cause of the disease, DNA from the proband was used to generate a 41.2-fold average-coverage whole genome sequence. This sequence contained 24,818 called variants relative to the canine reference sequence that were predicted to alter the primary structure of the encoded gene products. The proband was homozygous for 5811 of these variants. The proband’s homozygous variants were sorted according to the allele frequency among all 335 canine whole genome sequences included in the analysis. Within this cohort, variants in 12 genes were uniquely homozygous in the proband (Supplementary File S1). Among these was a C to T substitution at position 32,185,406 on chromosome 30, which was predicted to disrupt the intron 3–exon 4 splice site of *CLN6* (Figure 14). The validity of this variant call was confirmed by an Integrative-Genomics-Viewer-assisted inspection of aligned reads from the proband’s whole genome sequence to the Tasha reference sequence surrounding position 32,185,406 on chromosome 30 (Figure 15) and by the Sanger sequencing of the region surrounding the variant position. The disease phenotype was consistent with phenotypes previously associated with other *CLN6* variants [13,14], whereas a similar disease phenotype has not been associated with mutations in any of the other 11 genes that were uniquely homozygous in the proband (Supplementary File S1). The proband did not have potentially deleterious sequence variants in any of the other known NCL genes.

An allelic discrimination assay that distinguishes the reference and variant alleles at position 30: 32,185,406 was performed on 13 mixed-breed dogs with a variety of neurological disorders of unknown etiology. All 13 dogs were homozygous for the reference allele. This indicates that the *CLN6* variant is not a common cause of neurological disease in mixed-breed dogs.

**Figure 16:**
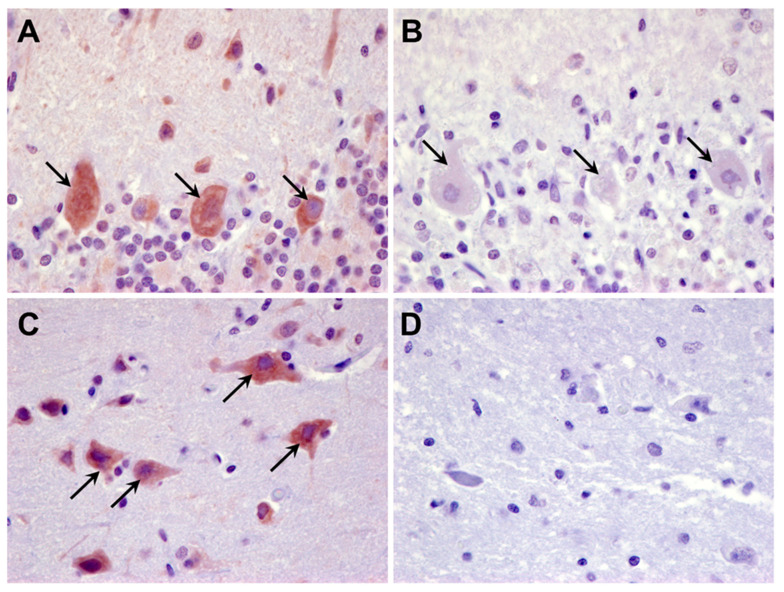
Paraffin sections of the cerebellar cortex (**A**) and cerebral cortex (**C**) of an unaffected Shiba Inu dog and the cerebellar cortex (**B**) and cerebral cortex (**D**) of the proband immunolabeled for the localization of the CLN6 protein (dark brown stain). In the cerebellar cortex of the unaffected dog, cells in the Purkinje layer exhibited pronounced immunolabeling (arrows in (**A**)), which was not observed in cerebellar cortex cells of the proband (arrows in (**B**)). Likewise, cells throughout the cerebral cortex gray matter of the unaffected dog exhibited substantial immunolabeling (arrows in (**C**)) that was not observed in the cerebral cortex gray matter of the proband (**D**).

The *CLN6* sequence variant is predicted to result in the skipping of exon 4 during pre-mRNA processing, and thus, there is a lack of full-length CLN6 protein. To determine whether any CLN6 protein was made, tissue sections from the proband were immunolabeled with an antibody directed against amino acid residues 282 to 311 of the protein that are encoded by exon 7 of *CLN6*. Cells in the cerebellum and cerebral cortex of an unaffected Shiba Inu dog exhibited pronounced CLN6 immunolabeling that was not observed in these tissues from the proband (Figure 16).

## 4. Discussion

The disorder evaluated in this study included all of the features that have been used to classify diseases as NCLs: progressive neurological and behavioral signs, brain atrophy, and an intracellular accumulation of storage bodies that exhibit lipofuscin-like autofluorescence. The ultrastructural appearances of the disease-related storage bodies were similar to those of other NCLs, as was the presence of mitochondrial ATP synthase subunit c protein in the storage bodies. Based on the ultrastructural features of the storage body contents, they appear to have derived, at least in part, from mitochondrial membranes. However, there were tissue-specific differences in the ultrastructure of the storage body contents. This may reflect differences in the molecular composition of the storage body precursors in different tissues, including tissue-specific differences in mitochondrial molecular composition [15,16,17]. Like other NCLs and similar neurodegenerative disorders, the proband exhibited significant neuroinflammation. Based on the current consensus of most investigators in the field, human disorders classified as NCLs result from mutations in *PPT1*, *TPP1*, *CLN3*, *DNAJC5*, *CLN5*, *CLN6*, *MFSD8*, *CLN8*, *CTSD*, *GRN*, *ATP13A2*, *CTSF*, and *KCTD7* [4,18]. All of these are autosomal recessive traits except the autosomal dominant disorder resulting from *DNAJC5* mutations [19]. Canine NCLs have been associated with mutations in *PPT1*, *TPP1*, *CLN5*, *CLN6*, *MFSD8*, *CLN8*, *CTSD*, and *ATP13A2* [10,13,20,21,22,23,24,25,26,27,28,29,30,31,32,33,34,35]. In addition, NCL-like disorders in dogs have been associated with mutations in *ARSG* and *CNP* [5,6,7]. Among these genes, the only potential deleterious variant in the whole genome sequence of the proband was a homozygous C to T substitution at the intron 3–exon 4 splice site of *CLN6*. This mutation is predicted to result in the absence of full-length CLN6 protein, and the protein was not detected with immunohistochemistry in the proband’s tissues.

Numerous pathogenic mutations in *CLN6* have been reported in human subjects suffering from NCL [14,36,37,38,39,40,41,42,43,44]. Among these are splice site variants in introns 2 and 4 and missense variants in all seven exons [36,37,45]. A missense mutation in *CLN6* exon 7 was also associated with NCL in an Australian Shepherd dog [13]. Thus, it is not surprising that the splice site mutation in the proband resulted in NCL disease.

Of the canine NCL disease variants, all but one occurs only within a single breed or in a few similar breeds in which interbreeding is likely to have occurred. The only previous exception is a mutation in *CLN5*, which has been associated with NCL in Border Collies, Australian Cattle Dogs, a German Shepherd–Australian Cattle Dog mix, and a mixed-breed dog of unknown ancestry [26]. In general, one would expect homozygosity for disease alleles to be less common in mixed-breed dogs than in purebred dogs as the latter tend to be much more highly inbred. An exception would be if two closely related mixed-breed dogs were mated. The proband in this study does not appear to be such an exception. The Wisdom Panel genotyping analysis indicated that the proband had 40% heterozygosity, which is typical for mixed-breed dogs and higher than most purebred dogs. The degree of heterozygosity would be significantly lower if the parents of the proband were closely related. It is extremely unlikely that the same variant would have occurred de novo in gametes from both parents. Therefore, at least one of the variant alleles almost certainly arose in an ancestral generation. Thus, it appears that the *CLN6* variant in the proband could be widespread, although probably uncommon, in the mixed-breed dog population and may be present in one or more ancestral breeds. Of the ancestral breeds identified in the proband’s Wisdom Panel, no cases of NCL have been reported that have not been associated with other previously identified NCL mutations. Should mixed-breed dogs or dogs of any breed present with NCL-like signs similar to those of the proband, it would be reasonable to test for the *CLN6* variant identified in this study. In a small sample of 13 mixed-breed dogs with neurological disorders of unknown etiology, none had the *CLN6* splice site variant. However, screening for this variant in mixed-breed dogs with NCL-like signs is warranted.

The mechanisms by which a lack of functional CLN6 protein causes lysosomal storage body accumulation and neurodegeneration are not well understood. CLN6 is a membrane protein that has been localized to the endoplasmic reticulum [4,46,47,48]. Evidence suggests that among the functions of CLN6 may be the regulation of sphingolipid and glycerophospholipid metabolism [49], the mediation of the delivery of proteins to lysosomes [50,51], the prevention of protein aggregation [52,53,54], the mediation of protein secretion from cells [55], and the regulation of the lipid composition of lysosomes [56]. Impairment of any of these functions could result in a general impairment of lysosomal function and the accumulation of substrates of lysosomal degradative enzymes. However, this would not explain the specific accumulation of ATP synthase subunit c protein that occurs in CLN6 disease and some of the other NCL disorders. The ultrastructural findings in this study in conjunction with previous research suggest that most components of autophagocytosed mitochondria other than the subunit c protein are degraded normally in CLN6-deficient cells [57,58,59,60]. The subunit c protein is very hydrophobic and prone to aggregation, properties that may explain its resistance to degradation and specific accumulation in lysosomes. Evidence suggests that CLN6 may be required for normal turnover of the subunit c protein within mitochondria, and that the accumulation of the protein within lysosomes is secondary to impaired degradation of subunit c within the mitochondria. A selective impairment of mitochondrial subunit c degradation in CLN6 disease is supported by the fact that in the cardiac muscle of the proband, storage material accumulated specifically in the mitochondria-rich perinuclear regions of the muscle fibers. In ovine CLN6 disease, it was found that excess subunit c protein selectively accumulates within the mitochondrial inner membrane, indicating that the disease results in impaired turnover of this protein within mitochondria [61]. This suggests that in addition to its other putative roles in cellular metabolism, the CLN6 protein may be important for maintaining the normal proteolytic degradation of subunit c within mitochondria and preventing secondary accumulation in lysosomal storage bodies [62,63]. The impaired turnover of subunit c within mitochondria may, in turn, result in the impairment of mitochondrial function that could play an important role in the CLN6 disease pathogenesis [64,65,66]. For example, a lack of normal CLN6 results in increased levels of the mitochondrial antioxidant enzyme manganese-dependent superoxide dismutase, suggesting an increased production of reactive oxygen species in mitochondria that can cause cell damage and death [67]. The evidence that a lack of normal CLN6 protein results in impaired subunit c protein turnover within mitochondria warrants research into whether, in addition to its localization in the endoplasmic reticulum, the CLN6 protein is also present in mitochondria, at least in the brain, retina, and cardiac muscle.

## Data Availability

DNA sequence data for the proband have been archived and deposited in the NCBI Sequence Read Archive as BioSample SAMN39309507.

## Supplementary Materials

The following supporting information can be downloaded at https://www.mdpi.com/article/10.3390/genes15060661/s1, Supplementary File S1: Sequence variants uniquely homozygous in proband relative to a 334 dog cohort. Ref. [68] is cited in Supplementary Materials.
