# Supplementary material for: Neuronal Ceroid Lipofuscinosis in a Mixed-Breed Dog with a Splice Site Variant in CLN6
Source: Genes (Basel). 2024 May 23;15(6):661. doi: 10.3390/genes15060661 (PMC11203140; doi:10.3390/genes15060661)
Supplement: Supplementary file 1 [file genes-15-00661-s001.zip › genes-3015157-supplementary.pdf]

Supplemental File 1

Sequence variants uniquely homozygous in proband relative to a 334 dog cohort

Gene	Predicted Protein Difference from Reference	Probability Variant will Affect Protein Function (SnpEFF prediction)*	Predicted Variant Effect on Protein Function (MutPred 2 prediction)*
CLN6	Exon 4 skipping	High	Deleterious**
TDRD5	V111I	Moderate	Neutral/non-deleterious
MASP1	G103S	Moderate	Neutral/non-deleterious
SLC41A1	Start gained	Low	Not assessed
FLNA	S1411L	Moderate	Neutral/non-deleterious
NPL	A298V	Moderate	Neutral/non-deleterious
KIF26B	K453R	Moderate	Neutral/non-deleterious
DSC1	K722N	Moderate	Potentially deleterious/moderate confidence
FREM1	T1265N	Moderate	Potentially deleterious/moderate confidence***
FREM1	T962R	Moderate	Likely deleterious***
LAMC2	R851Q	Moderate	Neutral/non-deleterious
SRCIN1	G721D	Moderate	Neutral/non-deleterious
PLEKHD1	Start gained	Low	Not assessed
IRS4	A1189AAA	Moderate	Neutral/non-deleterious

*Effect predictions based on SnpEff (https://pcingola.github.io/SnpEff/) and MutPred2 and MutPredindel (http://mutpred2.mutdb.org/index.html). Accessed 04/20/2024)

**Of the genes listed, *CLN6* is the only for which mutations have been reported to cause disorders similar to that exhibited by the proband.

****FREM1* encodes a basement membrane protein. Mutaions on *FREM1* cause disease phenotypes that are not similar to the disorder exhibited by the proband in this study [68].
